# Cyclosporin A Inhibits Rotavirus Replication and Restores Interferon-Beta Signaling Pathway *In Vitro* and *In Vivo*

**DOI:** 10.1371/journal.pone.0071815

**Published:** 2013-08-21

**Authors:** Zigang Shen, Haiyang He, Yuzhang Wu, Jintao Li

**Affiliations:** Institute of Immunology, PLA, Third Military Medical University, Chongqing, PR China; Temple University School of Medicine, United States of America

## Abstract

Rotavirus (RV) is the most common cause of severe diarrhea among infants and young children. Currently, there is no specific drug available against rotavirus, largely due to the lack of an ideal target molecule which has hampered drug development. Our previous studies have revealed that cyclosporin A (CsA) might be potentially useful as an anti-RV drug. We therefore used both cellular and mouse models to study the immunological safety and effectiveness of CsA as an anti-RV drug. We found that CsA treatment of HT-29 cells before, during, and after viral infection efficiently inhibited Wa strain RV replication and restored IFN-β expression in a HT-29 cell line model. Exploring the underlying mechanisms showed that CsA promoted Interferon Regulatory Factor-5 (IRF-5) expression (a key positive regulator of the type I IFN signaling pathway), but not IRF-1, IRF-3, or IRF-7. Additionally, CsA inhibited SOCS-1 expression (the key negative regulator of IFN-α/β), but not SOCS-2 or SOCS-3. The antiviral effect of CsA was confirmed in an RV-infected neonatal mouse model by evaluation of antigen clearance and assessment of changes in intestinal tissue pathology. Also, no differences in T cell frequency or proliferation between the CsA- and vehicle-treated groups were observed. Thus, both our in vitro and in vivo findings suggest that CsA, through modulating the expression of key regulators in IFN signaling pathway, promote type I IFN-based intracellular innate immunity in RV host cells. These findings suggest that CsA may be a useful candidate to develop a new anti-RV strategy, although further evaluation and characterization of CsA on RV-induced diarrhea are warranted.

## Introduction

Rotaviruses (RVs) are the chief etiologic agents of viral gastroenteritis in the young of a large variety of animal species, including human infants and young children. Acute diarrhea caused by RV represents a global health problem: RV causes 114 million episodes of diarrhea, resulting in 24 million clinic visits and 2.4 million hospitalizations annually [1]. Worldwide, rotavirus infection results in approximately 500,000 deaths; this equates to approximately 37% of all deaths and 5% of deaths in children <5 years old is attributable to diarrheal disease [2]. At present, two vaccines namely Rotarix® (GlaxoSmithKline) and RotaTeq (Merck) mediate an anti-diarrheal effect *in vivo* [3], [4]. However, they are neither globally distributed nor highly effective in some developing countries. To date, no specific anti-rotavirus drug is available, largely due to a lack of understanding of the molecular mechanisms underlying rotavirus-induced diarrhea. Therefore, there is a great need for improved vaccines and other anti-rotavirus treatments, such as specific rotavirus inhibitors, to prevent severe rotavirus-induced disease.

Cyclosporin A (CsA) has long been known to be a powerful immunosuppressive agent. It has been used to prevent rejection in kidney, liver, bone marrow, and pancreas organ transplant recipients. More recently studies have also shown its usefulness in other clinical applications, such as treating autoimmune disorders and viral diseases [5]. CsA was recognized as a promising antiviral agent because of its potential ability to inhibit hepatitis C virus (HCV) replication both *in vivo* and *in vitro* via a cyclophilin-dependent pathway [6], [7], [8]. CsA also inhibits hepatitis B virus (HBV), mouse cytomegalovirus virus (MCV), and human immunodeficiency virus (HIV) infections [9], [10], [11]. In contrast, CsA has been shown to promote influenza virus infection [12]. Our previous study showed that cyclophilin A (CYPA) transiently increases during rotavirus infection [13]. We found that CYPA was also critical for IFN-β production in the infection of natural virus like RV. We also showed that CsA, the CYPA Prolyl isomerase (also known as peptidylprolyl isomerase or PPIase) inhibitor, can restore IFN-β production [14]. Thus we hypothesized that CsA may be able to suppress rotavirus replication through IFN-β signaling pathway, and therefore reduce diarrhea.

Rotavirus infection induces host innate cellular defense mechanisms, including type I interferon (IFN) production in humans and animals [15], [16], which is crucial for controlling viral infection. Indeed, IFNs have been used as anti-rotavirus agents [17], [18], and the mechanisms underlying interferon induction are relatively well understood [19]. Following infection, the host recognizes viral components and activates IFN-regulatory factors (IRF), subsequently increasing type I IFN expression. Secreted type I IFNs bind to the type I IFN receptor (IFNAR) on surrounding uninfected cells, phosphorylating JAK1 and Tyk2 kinases that activate transcription factors STAT1 and STAT2 to form the heterotrimeric transcription factor complex called Interferon-stimulated gene factor 3 (ISGF3), which translocates to the nucleus and induces the expression of hundreds of IFN-stimulated genes (ISGs) with antiviral properties to establish an antiviral state within the host [19]. In turn, viruses have evolved many mechanisms in order to escape such host immunity [16], such as blocking IFN-α/β and ISG expression. Both simian (RRV) and human (Wa) rotavirus strains suppress IFN-α– and IFN-β–stimulated gene expression in MA104 (kidney epithelial) and Caco-2 (colonic epithelial) cell lines [20]. CsA was recently found to restore IFN-α expression in hepatocytes [21]. Therefore, it is plausible that CsA may induce host resistance to rotavirus infection by restoring IFN-α and/or IFN-β expression.

Cell-culture and animal models of rotavirus infection are useful in vaccine development and in identifying other drug treatments that may inhibit the virus. Taking all of these observations into account, we investigated whether CsA inhibits rotavirus replication in human colonic HT-29 cells and in a rotavirus-infected neonatal mouse model. We also investigated whether CsA is able to restore type I IFN expression in HT-29 cells. Additionally, we explored the mechanism of action that CsA exerts on Wa rotavirus and intracellular innate immunity at both the cellular and molecular levels. Our data revealed that CsA efficiently inhibits Wa rotavirus replication in HT-29 cells and in a rotavirus-infected neonatal mouse model, and restores IFN-β expression in HT-29 cells. These novel findings suggest that developing intervention strategies to reconstitute intracellular innate responses is of great interest to control rotavirus replication. Furthermore, this is the first report to show that CsA can suppress rotavirus infection.

## Materials and Methods

### Ethics Statement

All mouse work was done according to the requirements of Third Military Medical University Animal Ethics Committee approval number TMMU 08-08-01. Animals were sacrificed using CO_2_ asphyxiation and the appropriate organs harvested.

### Reagent and Virus Strains

CsA and Ribavirin were purchased from Sigma-Aldrich Inc. (St. Louis, Missouri, USA). Goat anti-Rotavirus immunofluorescence antibody was purchased from ViroStat Inc. (Po.Box, Portland, ME). Human Rotavirus antigen ELISA kit was obtained from DRG International Inc. (Mountainside, New Jersey, USA). The human RV Wa strain and kidney MA104 cells were kindly gifted by Dr. Elschner (Federal Institute for Health Protection of Consumers and Veterinary Medicine, Germany). Simian rotavirus SA11 strain was originally obtained from H. Malherbe (University of Texas Health Science Center, San Antonio, Tex). Human colon cancer HT-29 cells were bought from American Type Culture Collection (ATCC® HTB-38™).

### Cell Culture and RV Infection

MA104 cells and HT-29 cells were grown in Dulbecco’s-modified Eagle’s medium (DMEM)-10% FCS at 5% carbon dioxide. For all experiments, functionally endotoxin-free media and reagents were used. To prepare virus stocks, Wa and SA11 rotavirus were pretreated with crystalline trypsin (10 µg/mL) for 60 minutes at 37°C respectively. Subsequently, MA104 cells were infected at a multiplicity of infection (MOI) of 3, and harvested at 24–48 hours post-inoculation, when 80–95% cytopathic effect (CPE) was observed. Culture media was cleared of cell debris by centrifugation at 2000×g for 10 minutes, and the virus in the supernatant was concentrated by centrifugation at 80,000×g in an SW28 rotor for 2 hours at 4°C. Virus pellets were suspended in TNC buffer and stored in aliquots at –70°C.

### Lactate Dehydrogenase (LDH) Assay

LDH assay was conducted to measure CsA’s cytotoxicity on HT-29 cells, according to the manufacturer’s instructions using a LDH assay kit (Roche, Beijing, China).

### Titration of Extracellular and Intracellular Wa Rotavirus

The infectivity titer of Wa rotavirus was determined on HT-29 cells by the end-point dilution and immunofluorescence assay. When HT-29 cells were seeded in 12-well plates (10^5^ cells/well) for 24 h, the Wa rotavirus (MOI = 3) infected cells were treated with CsA at different concentrations at 12 h post-infection. Cell lysates and culture supernatants were collected at 24 h post-treatment. For titration of extracellular virus, the infected cell culture supernatant was serially diluted 10-fold in DMEM and used to infect HT-29 cells (10^4^ cells/well) in 96-well plates. Wa rotavirus infectivity was examined 24 hours post-infection by immunofluorescence assay. The viral titer is expressed as focus-forming units per milliliter of supernatant (FFU/mL). For titration of intracellular infection, rotavirus-infected HT-29 cells were washed once with PBS and subsequently incubated with 0.25% trypsin-EDTA for 3 minute at 37°C. Cells were resuspended in DMEM and collected by centrifugation at 1000 rpm for 3 minutes. The cell pellets were resuspended in DMEM and were lysed by 4 freeze-thaw cycles in dry ice and a 37°C water bath, respectively. Cell debris was pelleted by centrifugation for 5 min at 4000 rpm. The supernatants were collected and used for rotavirus infectivity titration.

### Enzyme-linked Immunosorbant Assay Analysis (ELISA)

Cell lysates and culture supernatants were collected at 24 h post-treatment. For detection of extracellular virus antigen, the infected cell culture supernatants were serially 10-fold diluted in DMEM and used to detect rotavirus antigen by ELISA kit (Jersey, USA) following the manufacturer’s instructions. For detection of intracellular virus antigen, rotavirus infected HT-29 cells were resuspended in DMEM and were lysed by 4 freeze-thaw cycles in dry ice and a 37°C water bath. The supernatants were collected and used for detection of rotavirus antigen, according to the manufacturer’s instructions. The test was considered positive if the optical density at 450 nm (OD450) of the well containing stool minus the OD450 of control wells was ≧ 0.1.

### Direct Immunofluorescence Staining

Immunofluorescence evaluation of rotavirus infection in HT-29 cells was carried out as previously described [22]. The infected HT-29 cells were treated with CsA and grown on coverslips in 24-well plates at 37°C. After washing with 1× phosphate-buffered saline(PBS), HT-29 cells were fixed with PBS containing 4% paraformaldehyde for 15 minutes, permeabilized with PBS containing 0.25% triton for 10 minutes, washed 4 times with PBS, and pretreated with a blocking solution for 30 minutes. Pretreated cells were air-dried, treated with goat anti-rotavirus immunofluorescence antibody in a humidity chamber for 1 hour at room temperature. The coverslip preparations were then washed with PBS, dried and covered with glycerine. The coverslip preparations were observed under a fluorescence microscope.

### Quantitative Real-time

Total cellular RNA extracted from HT-29 Cells was subjected to reverse transcription using the reverse transcription system from Toyobo (Osaka, Japan). Real-time PCR conditions using a LightCycler instrument to detect human rotavirus cDNA were optimized as described [23]. Briefly, Wa rotavirus RNA was extracted from 100 µL of the culture supernatant by TRI-Reagent-BD (Becton Drive Franklin Lakes, New Jersey, USA). Extracted RNA was then purified by an RNeasy Mini Kit and treated with RNase-free DNase digestion (Qiagen, Valencia, California, USA). The copy numbers of HRV RNA were determined by the Q-PCR in the specimen. The primers used in this study are listed in Table S1. The primers were synthesized by Sangon Biotech Co., Ltd. (Shanghai, China). The levels of glyceraldehyde-3-phosphate dehydrogenase (GAPDH) mRNA were used as an endogenous reference to normalize the quantities of target mRNA.

### Western Blotting

Cell lysates were harvested at the indicated times post-treatment with drugs in RIPA buffer (150 mM NaCl, 1% sodium deoxycholate, 1% Triton X-100, 0.1% SDS, 10 mM Tris-HCl pH 7.2), separated by SDS-PAGE, and transferred onto nitrocellulose membranes (Millipore). Primary antibodies against the following proteins were used: IRF-1 (Cell Signaling), IRF-3 (Santa Cruz), IRF-5 (Ambion), IRF-7 (Santa Cruz), SOCS-1 (Abcam), SOCS-2 (Abcam), SOCS-3 (Abcam) and GAPDH (Abcam). Primary antibodies were detected by using appropriate horseradish peroxidase-conjugated secondary antibody. Blots were developed by the chemiluminescent detection system from Pierce.

### Rotavirus Infection Mouse Model

Three-day-old specific pathogen-free BALB/c mice with their mothers were obtained from Animal Research centre of the Third Military Medical University (Chongqing, China). Animals were raised in filter-topped cages on a standard rodent chow diet with water available ad libitum. The experiments were performed according to national regulations and approved by the local animal ethics committee. Three-day-old mice were inoculated with SA11 rotavirus (10^7^ PFU) through oral gavages. Fecal samples were collected from each mouse at 0 to 3 days post infection, and the severity of diarrheal illness was assessed by examination of fecal material as described [24]. Mice from which no stool could be obtained were considered as mice with no diarrhea. Clinical score ≧ 2 was considered to be a symptom of diarrhea.

### Drugs Inoculations and Subsequent Animal-handling Procedures

The mice scored for diarrhea were randomly divided into 3 groups (30 mice each group) and ear coded. The mice were subjected to treatments with CsA (2.5 mg/kg/d) or Ribavirin (5 mg/kg/d) or PBS (Curing group, Control) by oral gavages. Each mouse’s healing time was recorded. Fecal samples were collected at 3 days of treatment, and detected rotavirus antigen using an ELISA kit following the manufacturer’s instructions. The ELISA cutoff value is 0.1 and test was considered positive if the optical density at 450 nm (OD450) of the well containing stool was ≧ 0.2. Stool rotavirus antigen clearance rate was calculated by dividing the number of rotavirus antigen-negative mice by the total number of mice.

### Histologic Examination

Mouse small intestine (jejunum) was dissected after 3 days of treatment (5 mice each group, 2 sections each mouse), fixed in 4% (w/v) paraformaldehyde and embedded in paraffin. The slices (5 µm) were cut, deparaffinized by being immersed in xylene and rehydrated in graded ethanol solutions as previously described [25]. The slices were stained by hematoxylin-eosin (HE) and observed under light microscope. The crypts depth and villi height were measured with software Image Pro Plus 5.1 (IPP5.1).

### Flow Analysis

For flow cytometry analysis, splenocytes, draining lymph node cells, thymus cells, were directly fixed and surface-stained (for CD3, CD4, CD8). Samples were acquired on a BD FACS CantorII machine and analyzed by using FlowJo software.

### CFSE Labeling Protocol

The CFSE labeling protocol has been described previously [26]. Briefly, single-cell suspensions from the spleen were counted and stained with CFSE at 5 µM at room temperature in the dark with continuous rocking. After 5 min, staining was quenched with heat-inactivated FBS and the cells were washed, counted, resuspended in complete medium, and used for in vitro cultures.

### ELISAs

IL-4, IL-2, and IFN-γ concentrations in culture supernatant fluids were determined by sandwich ELISA using the following antibody pairs from BioLegend (San Diego, CA):IL-2, JES6-1A12 and JES6-5H4, IFN-γ, R4-6A2 and XMG1.2; and IL-4, 11B11 and BVD6-24G2.

### Statistical Analysis

Where appropriate, data were expressed as mean ± standard deviation (SD) of triplicate cultures. Statistical significance was assessed with two-tailed Student’s t-test between two groups and by one-way ANOVA for comparisons among multiple groups. Statistical analyses were performed with Statistical Product and Service Solutions (SPSS) Windows13.0 Stastistical Software. Statistical significance was defined as P<0.05.

## Results

### CsA Inhibits Wa Rotavirus Replication

**Figure 1:**
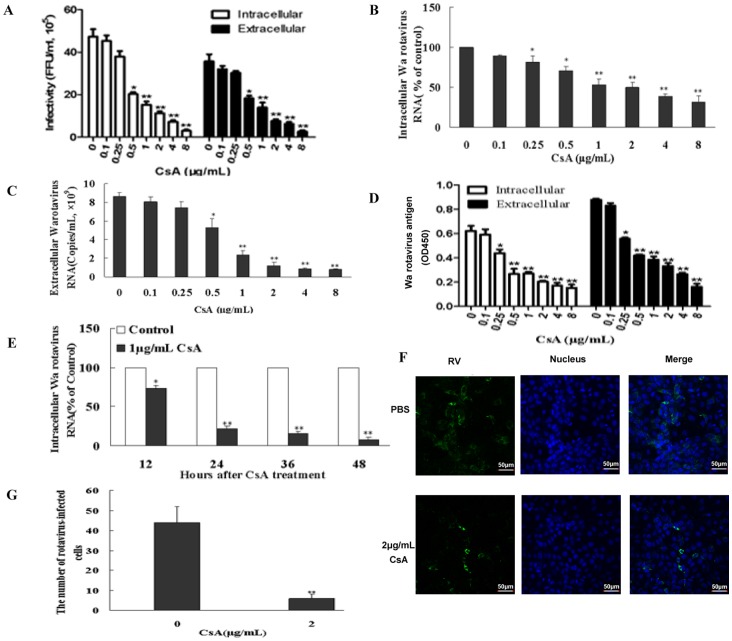
Effect of cyclosporin A (CsA) on Wa rotavirus replication *in vitro*. Infectious viral titers (A) were determined by serial dilution and immunofluorescence analysis. The intracellular or extracellular Wa rotavirus is expressed as focus-forming units per milliliter (FFU/mL) of cell lysate or supernatant, respectively. Wa rotavirus RNA expression levels (B) were determined by qRT-PCR. The levels of intracellular Wa rotavirus RNA are expressed as % of control (without CsA treatment, which is defined as 100) normalized to glyceraldehyde-3-phosphate dehydrogenase (GAPDH) mRNA. The levels of extracellular Wa rotavirus RNA (C) are expressed as viral copies/mL. Wa rotavirus antigen (D) levels were determined by ELISA. The intracellular or extracellular Wa rotavirus antigen is expressed as OD450. (E) Time-course effect of CsA on Wa rotavirus replication. Intracellular RNA levels, normalized to GAPDH mRNA, are expressed as % of control (without CsA treatment, which is defined as 100). (F) Immunofluorescent evaluation of rotavirus infection in HT29 cells was carried out by direct immunofluorescence staining with goat anti-rotavirus immunofluorescence antibody (green). (G) Histogram of immunofluorescence results. One representative experiment is illustrated (magnification: 200×). The results (A-G) shown are expressed as mean ± standard deviation of triplicate cultures, and 3 independent experiments were carried out (*P<0.05, **P<0.01).

To assess whether CsA treatment could inhibit rotavirus infectivity, HT-29 cells were infected with Wa rotavirus for 12 h and then treated with various CsA doses (0.1–8 µg/mL) for 24 h. Total cell lysates were collected to assess infectious viral titers by serial dilution and immunofluorescence analysis. The intracellular or extracellular Wa rotavirus is expressed as focus-forming units per milliliter (FFU/mL) of cell lysate or supernatant, respectively. We found a statistically significant reduction in infectivity by CsA treatment in a dose-dependent manner (Fig. 1A). This effect was not due to CsA-induced cytotoxicity of the host cells, as CsA induced very little cytotoxicity in HT-29 cells at <16 µg/mL doses in an LDH assay (data not shown). In order to confirm that CsA inhibited rotavirus infectivity, we also evaluated rotavirus antigen by ELISA and rotavirus RNA expression by qRT-PCR. Consistent with viral titer results, CsA treatment significantly reduced rotavirus RNA and antigen levels (Fig. 1B, C, D). By evaluating RNA levels, we found that CsA inhibited Wa rotavirus replication in a time-dependent manner (Fig. 1E). A direct immunofluorescence staining assay further confirmed that CsA suppressed Wa rotavirus replication in HT-29 cells compared to control (Fig. 1F, G).

**Figure 2:**
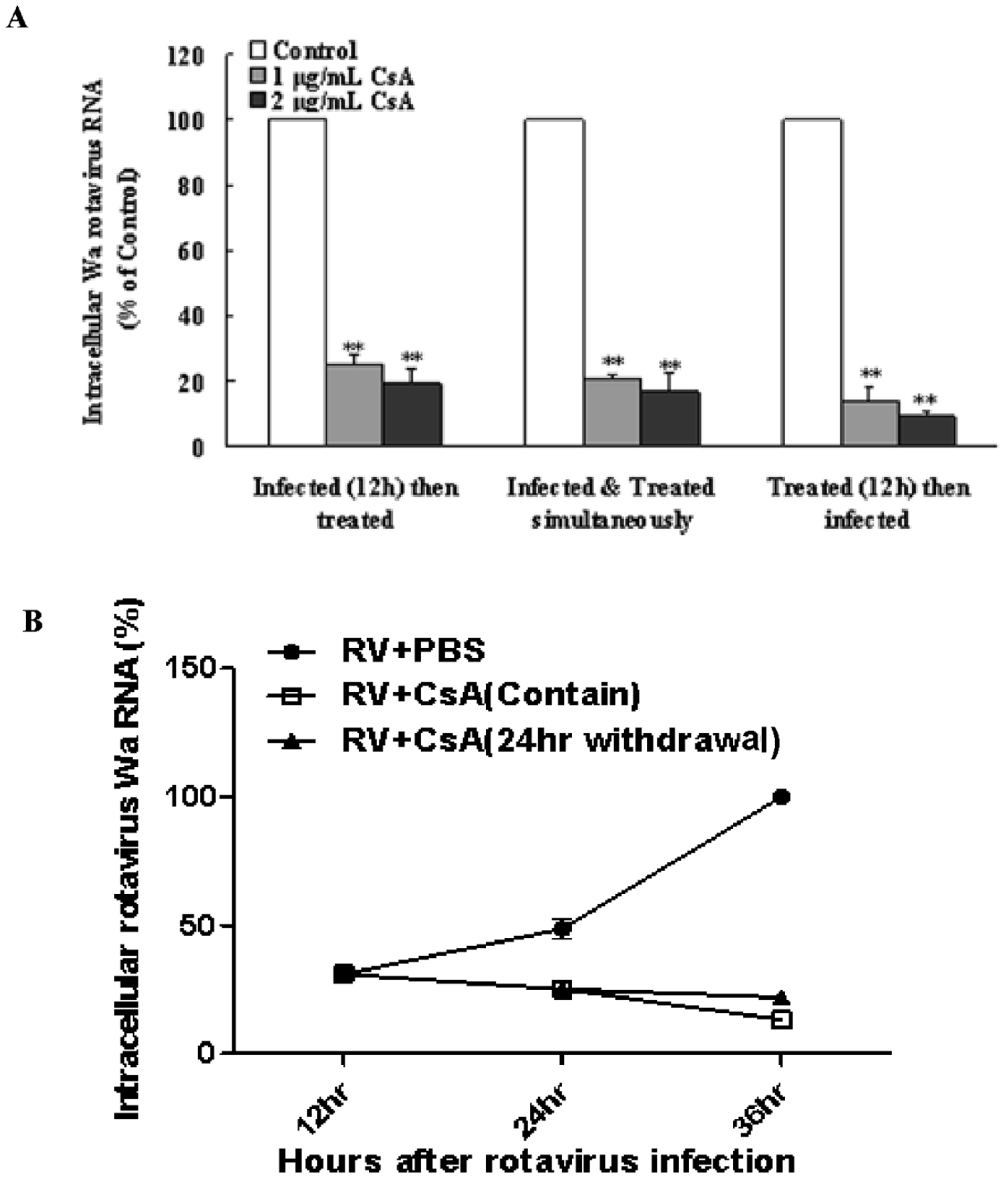
Effect of cyclosporin A (CsA) on Wa rotavirus infection and replication under various conditions. (A) Effect of different CsA treatment conditions on Wa rotavirus infection HT-29 cells, as follows: CsA treatment first for 12 h followed by Wa rotavirus infection for 24 h; simultaneous CsA treatment and Wa rotavirus infection followed by 24 h culture; or Wa rotavirus infection first for 12 h followed by CsA treatment for 24 h. Total cellular RNA extracted from the cell cultures was subjected to RT-PCR to quantify Wa rotavirus and GAPDH RNA. Intracellular Wa rotavirus RNA levels, normalized to GAPDH mRNA, are expressed as % of control (without CsA treatment, which is defined as 100). (B) Effect of CsA treatment withdrawal on Wa rotavirus replication. Control: HT-29 cells were infected with Wa rotavirus without CsA treatment; Maintain: Wa rotavirus-infected HT-29 cells were treated with CsA for 12 h, then cells were then cultured for additional 12 h in the presence of CsA; Withdrawal: Wa rotavirus-infected HT-29 cells were treated with CsA for 12 h, then cells were then cultured for additional 12 h in the absence of CsA. Intracellular Wa rotavirus RNA levels, normalized to GAPDH mRNA, are expressed as % of control (without CsA treatment, which is defined as 100 at 36 h post-infection). Data shown are expressed as mean ± standard deviation of triplicate cultures, and 3 independent experiments were carried out (*P<0.05, **P<0.01).

We next wondered whether the CsA-mediated antiviral effect is transient or permanent, and addressed this by investigating the effect of CsA on HT-29 cells under three different treatment conditions: before, during, and after rotavirus infection. All three different CsA treatments were able to suppress Wa rotavirus expression (Fig. 2A). In order to determine whether CsA-mediated suppression of Wa rotavirus expression in HT-29 cells could be maintained after initial treatment, we eliminated CsA, from HRV-infected cultures; this withdrawal did not lead to rebound of Wa rotavirus replication as compared to continual drug treatment (Fig. 2B).

### CSA Restores IFN-β Expression in Rotavirus-infected HT-29 Cells

**Figure 3:**
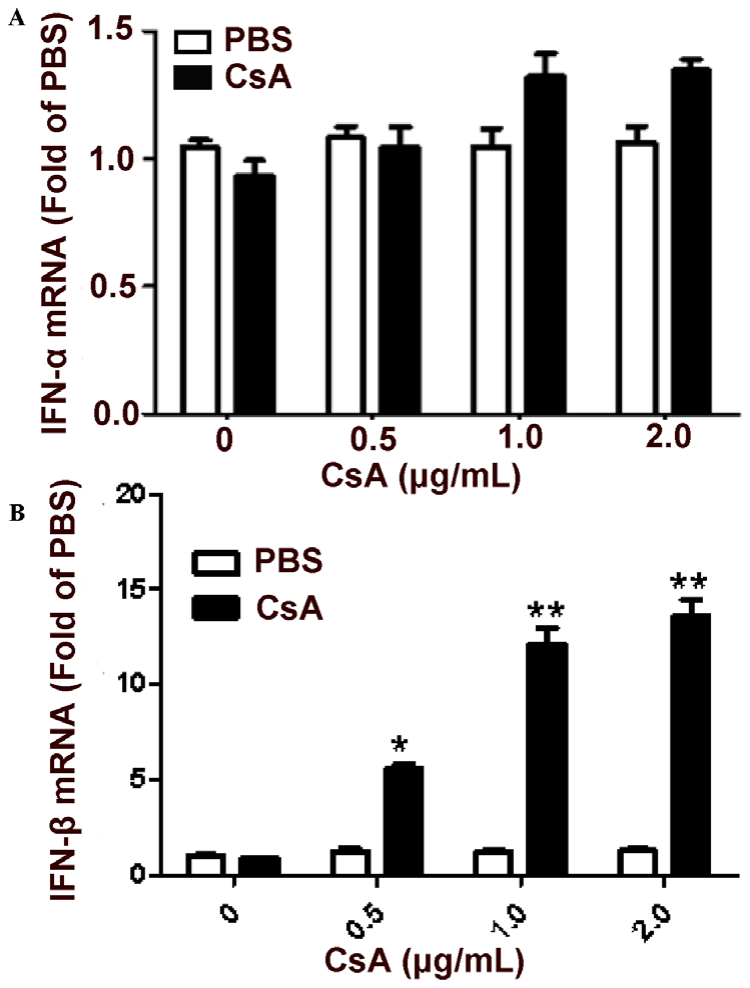
Effect of cyclosporin A (CsA) on type I interferon expression. Wa rotavirus-infected HT-29 cells were treated with CsA at indicated doses at 12 h post-infection. Total cell lysates were collected at 24 h post-treatment. Total cellular RNA extracted from the total cell lysates was subjected to the real-time RT-PCR to quantify IFN-α (A) and IFN-β (B). IFN-α/IFN-β expression is expressed as mRNA levels relative (fold) to the control (uninfected HT-29 cells without CsA treatment, which is defined as 1). Data shown are expressed as mean ± standard deviation of triplicate cultures, and 3 independent experiments were carried out (*P<0.05, **P<0.01).

Several rotavirus strains, including simian rotavirus SA11, human rotavirus Wa, and bovine rotavirus B641, have all previously been shown to suppress IFN production in infected cells through their own respective mechanisms [20], [27], [28]. We thus examined whether CsA repressed rotavirus replication by restoring type I IFN expression in Wa rotavirus-infected HT-29 cells. To this end, HT-29 cells were infected with Wa rotavirus for 12 h, treated with CsA for 24 h, and evaluated for type I IFN expression by qRT-PCR. While IFN-β expression was significantly increased in Wa-rotavirus-infected cells treated with CsA (Fig. 3B), CsA had little effect on IFN-α expression (Fig. 3A). These results provide further evidence that this drug treatment can restore type I IFN expression and increase antiviral immunity.

### CsA Modulates the Expression of IFN Pathway Regulators

**Figure 4:**
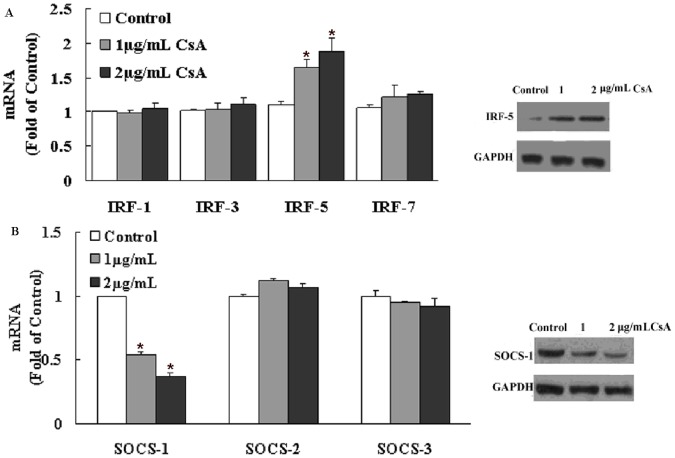
Effect of cyclosporin A (CsA) on the expression of the type I interferon (IFN) signaling pathway regulators. Wa rotavirus-infected HT-29 cells were treated with CsA at indicated concentrations at 12 h post-infection. Total cell lysates were collected at 24 h post-treatment. (A) Interferon regulatory factor (IRF)-1, -3, -5, and -7 mRNA levels were analyzed by real-time PCR, and protein levels were measured by western blotting. GAPDH was used as an internal control for RT-PCR and a loading control for western blotting. (B) Suppressor of cytokine signaling (SOCS)-1, -2, and -3 mRNA levels were analyzed by real-time PCR, and protein levels were measured by western blotting. GAPDH was used as an internal control for RT-PCR and a loading control for western blotting. Data are expressed as mRNA levels relative (fold) to the control (without CsA treatment, which is defined as 1). Data shown are expressed as mean ± standard deviation of triplicate culture, and 3 independent experiments were carried out (*P<0.05).

Viral infection in most cells induces type I IFN expression and secretion, which is controlled by multiple transcription factors. We first examined whether CsA could modulate the expression of key IFN pathway regulators by qRT-PCR. While CsA induced the expression of interferon regulatory factor 5 (IRF-5), IRF-1, IRF-3, and IRF-7 expression did not change (Fig. 4A). In addition, suppressor of cytokine signaling-1 (SOCS-1) expression, a protein inhibitor of activated signal transducers, was decreased in rotavirus-infected cells after CsA treatment (Fig. 4B). CsA regulation of IRF-5 and SOCS-1 was confirmed at the protein level, as their protein levels showed the same trend as the mRNA results as assessed by western blot (Fig. 4A, B). Other IFN pathway regulatory proteins such as.IRF1, IRF3, IRF7, SOCS-2 and SOCS-3 did not change after CsA treatment (Figure S1).

### CsA Reduced Healing Time after Diarrhea Onset in A Rotavirus-infected Neonatal Mouse Model

**Figure 5:**
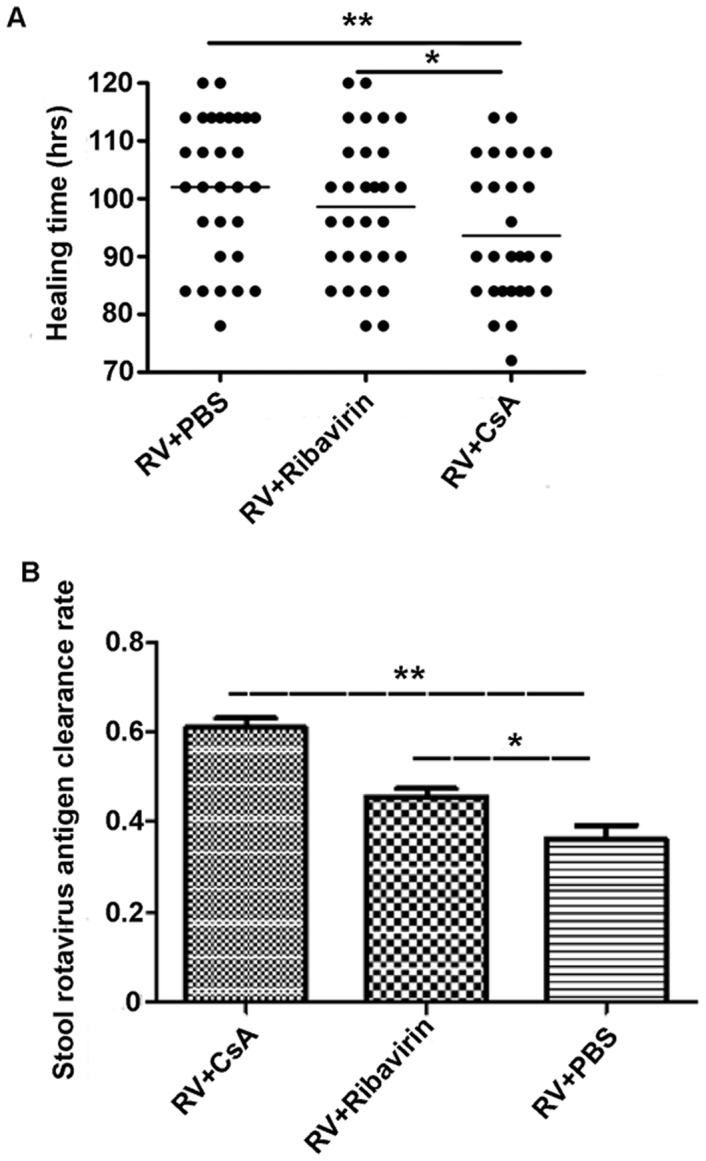
Effect of CsA on SA11 rotavirus-infected neonatal mouse model. Neonatal mice were inoculated with SA11 rotavirus, and mice developed diarrhea during the 18–24 h of experimental observation. The mice scored for diarrhea were randomly divided into 3 groups (30 mice each group). Mice were subjected to PBS, Ribavirin (5 mg/kg/d), or CsA (2.5 mg/kg/d) treatments by oral gavages. (A) Healing time. (B) Rotavirus-antigen clearance rate (%) in stool. Data shown are expressed as mean ± standard deviation of triplicate cultures, and are representative of 3 experiments (*P<0.05, **P<0.01).

**Figure 6:**
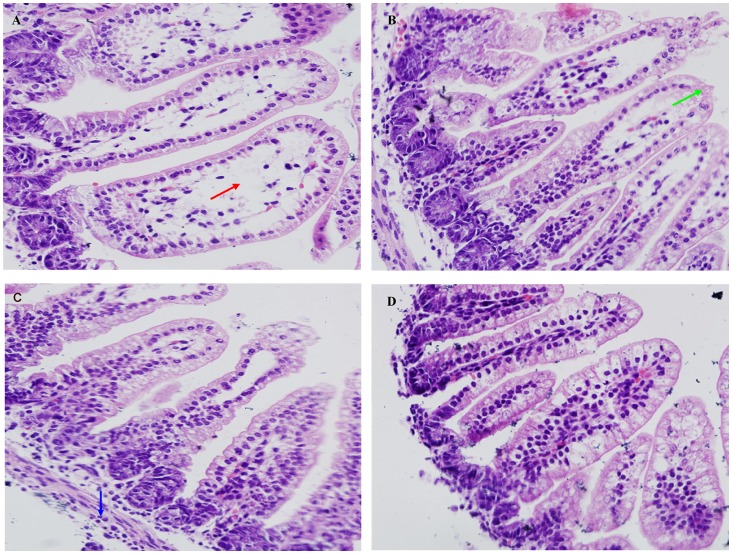
Jejunum changes as assessed by histology using a light microscope. (A) RV+PBS group. (B) RV+Ribavirin group. (C) RV+CsA group. (D) PBS group. (A-D) Morphologic changes in mouse jejunum pathology under a light microscope after 3 days of treatment (H&E, magnification: 200×). Red arrow: Villus vacuolar degeneration. Green arrow: Lesion on the epithelium. Blue arrow: Intestinal hydropsia.

Since CsA suppressed rotavirus replication *in vitro*, we further investigated whether CsA could affect rotavirus-induced diarrhea *in vivo*. Neonatal mice were inoculated with SA11 rotavirus and developed diarrhea during the following 18–24 h of experimental observation. Mean healing time in the CsA (93.6±8.16 hrs) and Ribavirin (used as an antiviral positive control) groups (98.64±6 hrs) were significantly shorter than in the control group (106.2±5.76 hrs) (Fig. 5A). After 3 days of treatment, the rotavirus-antigen clearance rate in stool from the CsA group increased by 27.4% compared to the control group (Fig. 5B). We next evaluated the pathology in the jejunum by histology. The lesions observed in the small intestine villi of the control group, including epithelium defluxion, dropsy, and vacuolar degeneration, were significantly reduced in the CsA-treated group (Fig. 6A, B, C, D). Taken together, our data highlights CsA as a potential therapeutic agent for RV infection.

Rotavirus mainly affects the small intestine villus epithelial cells, leading to the vacuolar degeneration, dropsy and defluxion of epithelial cells, which can be observed obviously in the epithelial cells of in RV infected neonatal mice (Fig. 6A), but not in PBS inoculated mice (Fig. 6D). We also observed that these effects were significantly attenuated in drug-treated groups (Fig. 6B, C). Three days after treatments, there was no significant difference of villi height among groups, but crypt cell reduction was observed in the Ribavirin and CsA groups compared to the control (Table S2), with the villi height and crypt depth measuring 313.54±18.25 µm and 30.19±1.22 µm, respectively, in the uninfected and untreated control group. Viewing the sections under an electron microscope, we observed that mice in the CsA group exhibited significantly reduced lipid droplet-like structures at the top of the intestinal cells that were abundant in control mice, although no obvious changes in microvilli misalignment and structure at the cell junctions were observed (data not shown). These data suggest that CsA treatment could not only reduce the healing time for diarrhea after rotavirus infection, but also mainly reduced the infection-induced pathology in the jejunum of infected mice.

### CsA Treatment does not Induce Long-term Immune Suppression in the RV-infected Mouse Model

**Figure 7:**
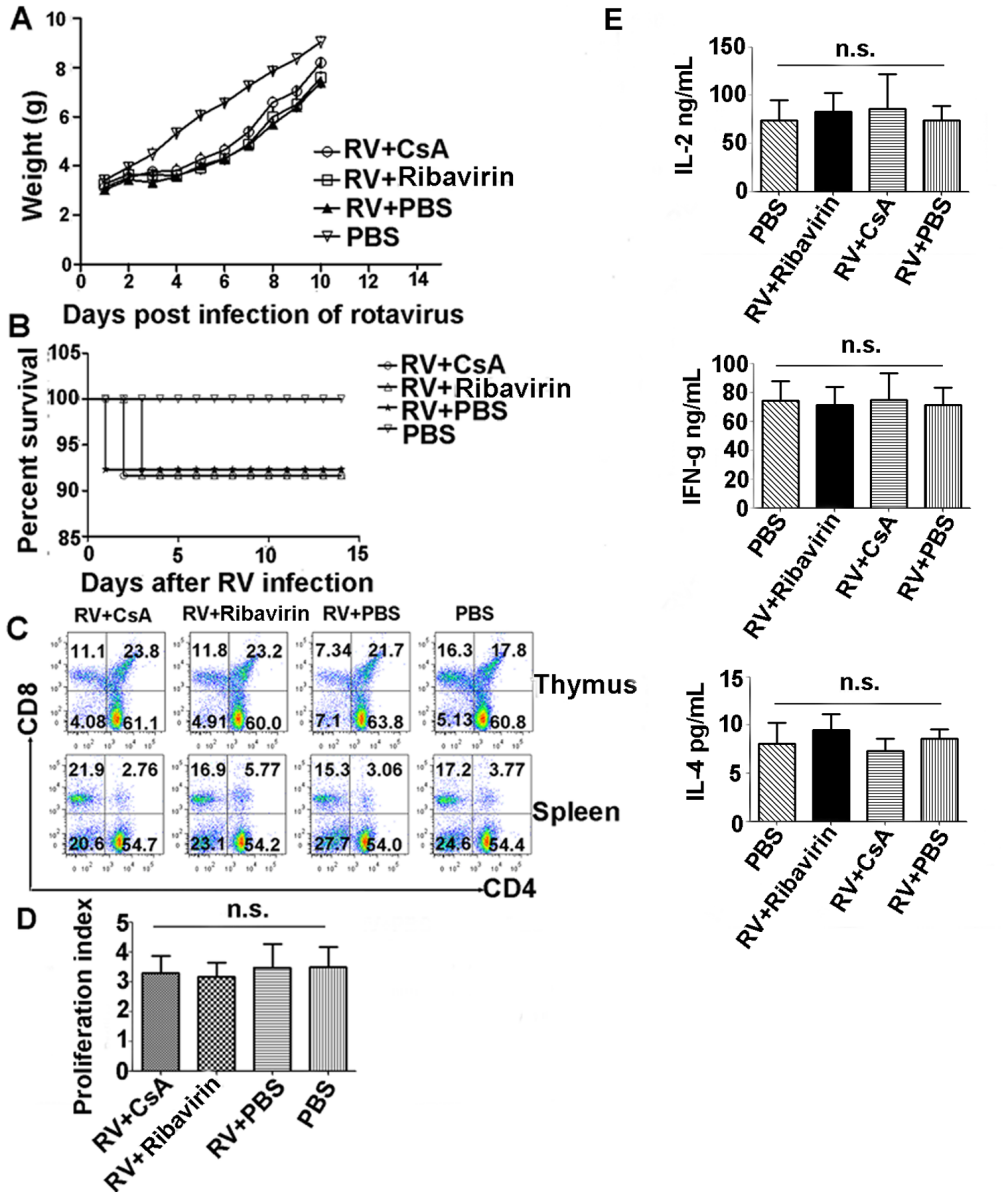
CsA toxicity analysis in the animal model. Body weight growth curve (A) and survival curve (B) after CsA treatment of RV-infected BALB/c mice. Body weight or survival were monitored among the 4 groups of mice during the week following rotavirus infection challenge CsA or PBS treatment (mean ± SEM, n >10). (C) CD4/CD8 T-cell ratio analysis in spleens and thymus from RV-infected mice treated with CsA or Ribavirin. (D) Proliferation assay of splenocytes in RV infected and CsA treatment mice by CFSE labeling. Ribavirin treatment mice as positive control and PBS treatment mice as negative control. Proliferation index (PI) was 3.47±0.37, 3.16±0.47, 3.47±0.79, 3.49±0.67 respectively. (E) IL-2, IL-4, and IFN-γ analysis from cell culture supernatants by sandwich ELISA. Data are representative of at least 3 independent experiments (n = 10 mice/group). Error bars indicate the SD with technical triplicates. N.s., not significant (*P<0.05, **P<0.01).

To analyze whether CsA treatment in this rotavirus-infected mouse model also induced immune suppression, the daily weight change, percent survival, T cell frequency in thymus and spleen cells, and T cell proliferation were evaluated. Except for rotavirus inoculation, non-infected control mice were otherwise simultaneously but separately reared under similar conditions. We first evaluated body weight changes after RV infection and CsA treatment and found that infected mice treated with CsA or Ribavirin neither gained nor lost body weight 1–4 days post-rotavirus infection (Fig. 7A), coincident with their rotavirus-induced diarrhea. Thereafter, infected mice resumed growth; the average daily gain for the mice in the CsA- and Ribavirin-treated groups during the 5–10 day period post-rotavirus infection (Fig. 7A) was similar to that of PBS-treated mice (*P = *0.1597 and *P = *0.0663, respectively, where weight values are expressed as mean ± standard error [SEM]). However, the average body weight of infected mice at the end of the experiment (10 days of age) was significantly lower (*P = *0.0472) than that of non-infected mice. We also evaluated survival among the CsA-, Ribavirin-, and PBS-treated groups as well as the uninfected group (Fig. 7B) and found no significant differences in their survival curves (*P = *0.7879).

To test the effect of the CsA dose on lymphocyte number in vivo, splenocytes and thymus cells were analyzed by flow cytometry for CD3, CD4, and CD8 expression. As shown in Fig. 7C, normal CD4 and CD8 T cell frequency and number were present in the CsA-treated group compared to the PBS control group. Considering that CsA is well known to suppress T cell proliferation [29], we determined whether this hindrance of T cell proliferation occurs in RV infected mice by labeling splenocytes isolated from CsA and PBS treatment groups with 5 µm CFSE and culturing them with non-specific stimulators. As shown in Fig. 7D, T cell proliferation by splenocytes in the CsA group was not different from PBS control group. Furthermore, Th1/Th2 cytokine polarization in cell culture supernatants was not different between groups as analyzed by ELISA, confirming the conclusion that CsA treatment does not induce immunosuppression in our rotavirus-infected mouse model (Fig. 7E).

## Discussion

Rotavirus infections occur in a great number of infants and young children worldwide. It can result in severe dehydrating gastroenteritis, often leading to hospitalization, and is occasionally associated with fatality [30]. Currently, however, no specific drug against rotavirus infection is available. We demonstrate here that CsA treatment of HT-29 cells *in vitro* suppresses Wa rotavirus infection, as reduced infectious viral titers were observed in rotavirus-infected HT-29 cells treated with CsA. Furthermore, CsA inhibited Wa rotavirus replication, as it decreased rotavirus RNA, antigen, and protein expression in HT-29 cells. CsA is also well known as a potent immunosuppressive drug, and large doses may cause kidney and liver toxicity, especially through the i.v. route [31], [32]. However, the CsA doses used in the study (ranging from 0.1–16 µg/mL) are not toxic to HT-29 cells, since they are below the <0.01 mM threshold found by LDH assay to have little cytotoxic effect on HT-29 cells. Additionally, treating cells with CsA before, during, or after rotavirus infection all resulted in significant inhibition of rotavirus replication. Taken together, these results suggest that CsA can inhibit rotavirus replication without causing host-cell cytotoxicity. Wa rotavirus has been reported to have the ability to block type I IFN synthesis [20], and most rotaviruses are known to share the ability to inhibit the IFN-β response [33]. Since CsA treatment can inhibit Wa rotavirus replication and infection, we further investigated whether this inhibition mechanism involved restoring type I IFN expression, and thus restoring an anti-viral state, in HT-29 cells. Since we found that IFN-β, but not IFN-α, expression was significantly increased, we speculate that CsA treatment restores IFN-β expression through suppressing Wa rotavirus and its proteins, not IFN-α and/or IFN-β regulatory genes. This view is supported by other reports about the role of HCV in regulating type I IFNs [34], [35]. Rotavirus inhibits type I IFN expression by interacting with nonstructural protein 1 (NSP1), which can bind to the IRF family and antagonize the IFN-signaling pathway to evade host immunity [33]. Since type I IFNs, IFN-β in particular, play a key role in host cell innate immunity against rotavirus infection, restoring IFN-β expression by CsA treatment could provide an antiviral environment.

Activation of the antiviral state and induction of type I IFN expression are directly modulated by the IRF family of transcription factors. Among the known IRFs, IRF3, IRF5, and IRF7 have been implicated as key positive regulators of type I IFNs [36], [37]; they not only recognize IFN promoter elements to selectively modulate type I IFN gene expression, but also regulate the IFN-stimulated response element (ISRE) in some IFN-stimulated genes (ISGs), leading to the induction of an antiviral state [37]. While rotavirus inhibits type I IFN expression and antagonizes cellular antiviral responses by inhibiting IRF function or the nuclear accumulation of STAT1, STAT2, and NF-κB [20], our data shows that CsA restores IFN-β expression in rotavirus-infected HT-29 cells. We therefore examined whether CsA affected the STAT-IFN pathway to modulate the IRFs. Indeed, CsA treatment significantly increased IRF-5 expression, which is known to support the development of antiviral responses in Wa rotavirus-infected HT-29 cells. To further explore the mechanisms underlying CSA-mediated enhancement of type I IFN expression, we examined whether CsA treatment inhibited the expression of negative regulators of the IFN signaling pathway, such as the SOCS suppressor family [38]. Among the SOCS molecules we evaluated, SOCS-1 was decreased after CsA treatment of rotavirus-infected cells, which may provide a sound mechanism to explain how CsA functions to restore IFN-β expression in rotavirus-infected cells.

It is known that cyclophilin A transiently increased during rotavirus infection in our previous work. We also found that CYPA transcription was decreased by siRNA thus increased RV replication [13]. In the present study, we surprisingly found that CsA, the CYPA PPIase inhibitor, can decrease the RV replication through increasing the IFN-beta levels. This means that the RV replication is a CYPA PPIase activity independent pathway. Furthermore, we have analyzed the CYPA expression in Wa rotavirus infected HT-29 cells treated with CSA, and found CYPA expression level was not significantly different (Figure S2). This result indicated that CsA can only inhibit the CYPA PPIase acitivity, but can’t affect the CYPA protein expression. Thus, we hypothesize that CsA plays a new role on CYPA to RV infection in an unknown molecule mechanism. So, we speculate that cyclosporine A inhibits the rotavirus virus replication through A CYPA PPIase independent pathway.

In this study, we also investigated the effect of CsA on rotavirus-induced diarrhea using a rotavirus-infected neonatal mouse model. Our data shows that CsA reduces the time it takes for mice to recover from rotavirus-induced diarrhea as compared to untreated groups (12 h reduction on average), and speeds up the elimination of rotavirus antigen from the host. Most convincingly, rotavirus-induced lesions in jejunum tissue were significantly improved in the small intestine after CsA treatment as compared to both the Ribavirin- and PBS-treated groups. Epithelial cell apoptosis promoted by rotavirus can lead to villous atrophy in the small intestine; however, we did not observe this phenomenon. It is possible that villous atrophy stimulates the proliferation of intestinal crypt cells. To support this view, we observed that the depth of the crypt cells were longer in PBS-treated group, compared to CsA-treated group. Rotavirus diarrhea may be induced by malabsorption and excessive secretion; while disorders in lipid metabolism and glucose metabolism can be associated with abnormal nutrient absorption. We observed that mice in the CsA-treated group exhibited significantly reduced lipid droplet-like structures, suggesting that lipid metabolism and glucose metabolism disorders in neonatal mice were partially recovered. Recent animal studies using CsA to reduce transplant rejection indicate that CsA can affect immune cell development, mainly affecting CD4^+^ T cells, especially to suppress CD4^+^ T cell proliferation [29]; our results showed that the CsA dose we used in rotavirus infected mice are safe and has no significant immunosuppressive effect compared to Ribavirin- and PBS-treated groups. Overall, these results indicate that further evaluation and characterization of this promising inhibitory effect of CsA on rotavirus-infected diarrhea are warranted.

## Supporting Information

Figure S1 **Effect of CsA on the expression of other IFN pathway regulatory proteins.** (A) Statistic analysis of band relative intensity of IRF-1, IRF-3, IRF-7, SOCS-2, SOCS-3. Error bars indicate the SD with technical triplicates. N.s., not significant. (B) Western blot of IRF-1, IRF-3, IRF-7, SOCS-2, SOCS-3 and GAPDH. GAPDH was used as an internal control.
(TIF)

Figure S2 **Time lapse expression patterns of CYPA in Wa rotavirus infected HT-29 cells with treated with CsA.** Wa rotavirus infected HT-29 cells were treated with CsA at indicated concentrations, and total cell lysates were collected at different times after treatment. CYPA protein level was measured by western blotting. GAPDH was used as an internal control.
(TIF)

Table S1 **Primes used for real-time reverse-transcription polymerase chain reaction assay.**
(TIF)

Table S2 **Villi height and crypts depth of jejunum post treatments with different drugs.**
(TIF)
