# Supplementary figures and images for: Cyclosporin A Inhibits Rotavirus Replication and Restores Interferon-Beta Signaling Pathway In Vitro and In Vivo
Source: PLoS One. 2013 Aug 21;8(8):e71815. doi: 10.1371/journal.pone.0071815 (PMC3749198; doi:10.1371/journal.pone.0071815)

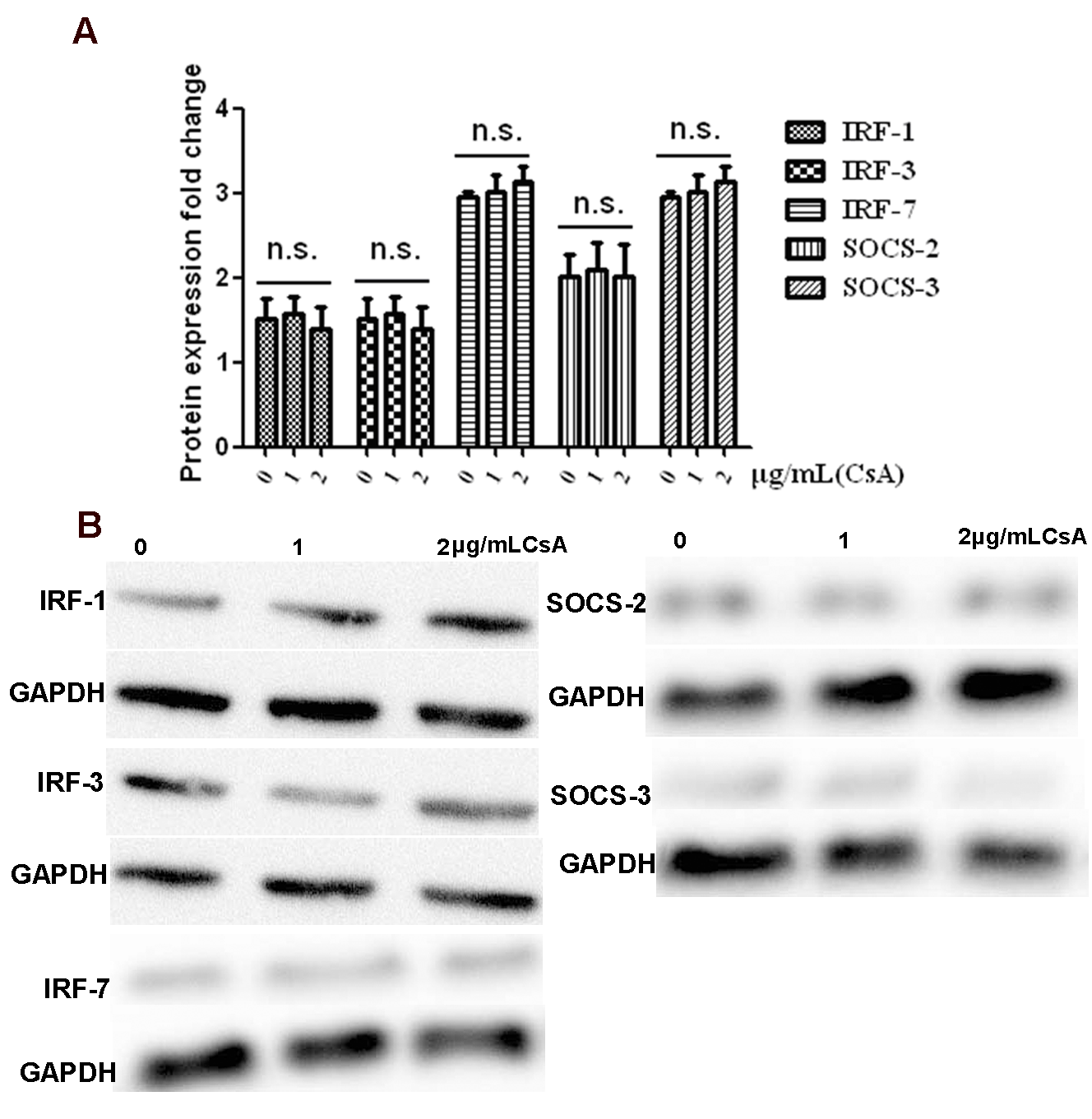

Supplement: Figure S1 — Effect of CsA on the expression of other IFN pathway regulatory proteins. (A) Statistic analysis of band relative intensity of IRF-1, IRF-3, IRF-7, SOCS-2, SOCS-3. Error bars indicate the SD with technical triplicates. N.s., not significant. (B) Western blot of IRF-1, IRF-3, IRF-7, SOCS-2, SOCS-3 and GAPDH. GAPDH was used as an internal control. (TIF) [file pone.0071815.s001.tif]

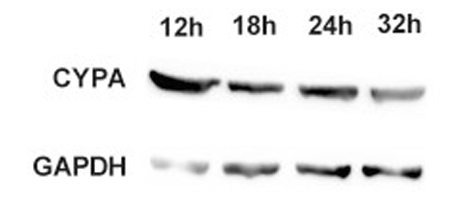

Supplement: Figure S2 — Time lapse expression patterns of CYPA in Wa rotavirus infected HT-29 cells with treated with CsA. Wa rotavirus infected HT-29 cells were treated with CsA at indicated concentrations, and total cell lysates were collected at different times after treatment. CYPA protein level was measured by western blotting. GAPDH was used as an internal control. (TIF) [file pone.0071815.s002.tif]

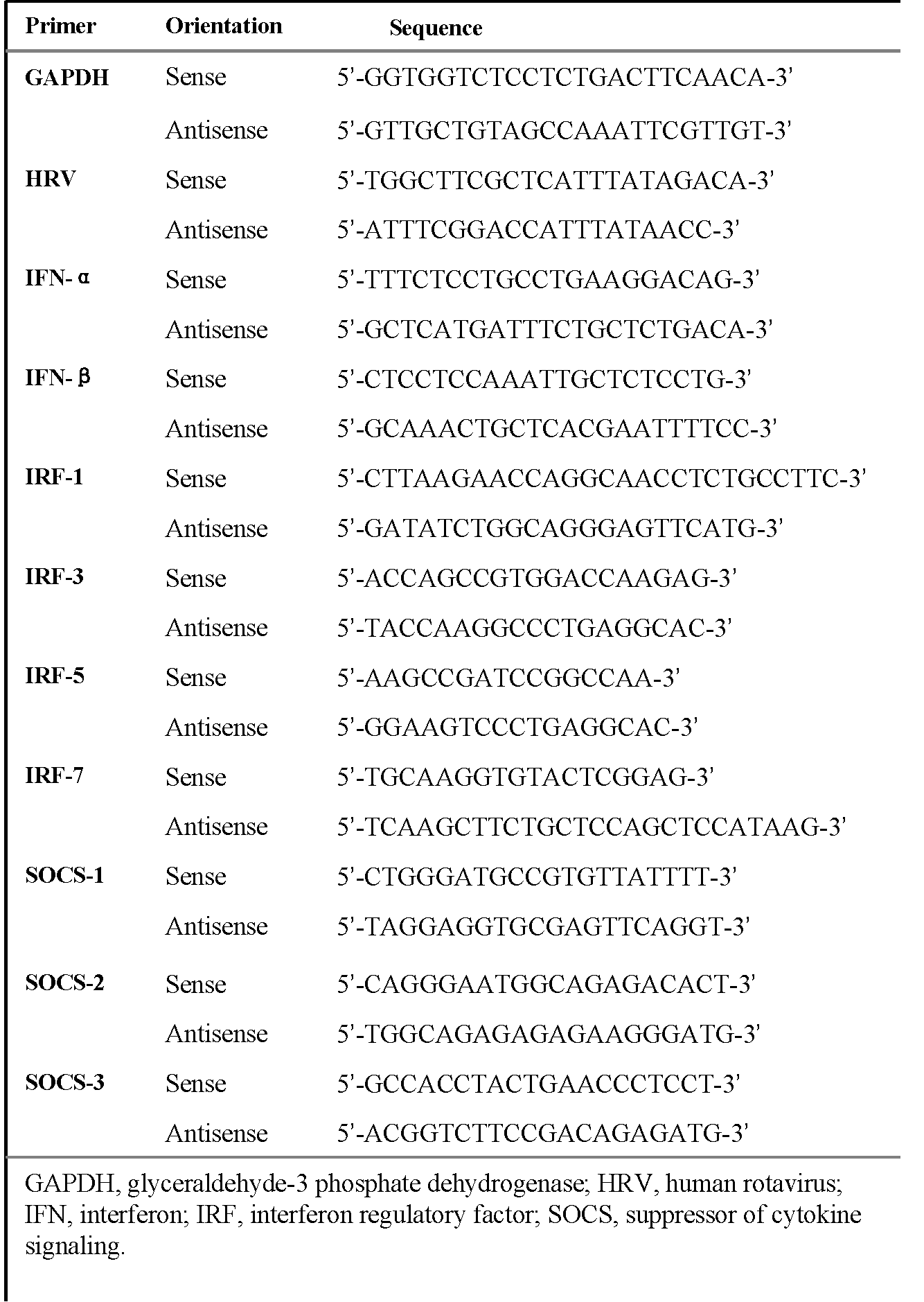

Supplement: Table S1 — Primes used for real-time reverse-transcription polymerase chain reaction assay. (TIF) [file pone.0071815.s003.tif]

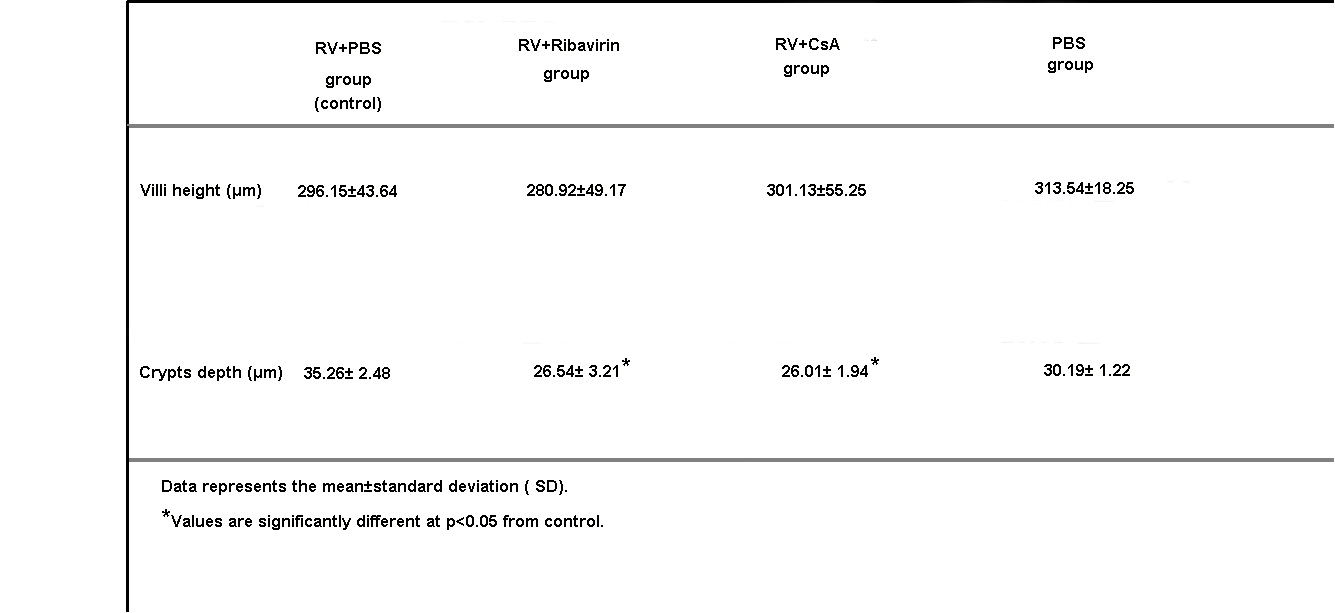

Supplement: Table S2 — Villi height and crypts depth of jejunum post treatments with different drugs. (TIF) [file pone.0071815.s004.tif]
